# Profiling the Structural Determinants of Aryl Benzamide Derivatives as Negative Allosteric Modulators of mGluR5 by In Silico Study

**DOI:** 10.3390/molecules25020406

**Published:** 2020-01-18

**Authors:** Yujing Zhao, Jiabin Chen, Qilei Liu, Yan Li

**Affiliations:** 1 Key Laboratory of Industrial Ecology and Environmental Engineering, Faculty of Chemical, Environmental and Biological Science and Technology, Dalian University of Technology, Dalian 116024, China; xiangrikui@mail.dlut.edu.cn (Y.Z.); 627391329@mail.dlut.edu.cn (J.C.); 2 Institute of Chemical Process Systems Engineering, School of Chemical Engineering, Dalian University of Technology, Dalian 116024, China; 201244335@mail.dlut.edu.cn

**Keywords:** mGluR5, NAMs, depression, 3D-QSAR, molecular docking, molecular dynamics, “arc” configuration

## Abstract

Glutamate plays a crucial role in the treatment of depression by interacting with the metabotropic glutamate receptor subtype 5 (mGluR5), whose negative allosteric modulators (NAMs) are thus promising antidepressants. At present, to explore the structural features of 106 newly synthesized aryl benzamide series molecules as mGluR5 NAMs, a set of ligand-based three-dimensional quantitative structure-activity relationship (3D-QSAR) analyses were firstly carried out applying comparative molecular field analysis (CoMFA) and comparative molecular similarity indices analysis (CoMSIA) methods. In addition, receptor-based analysis, namely molecular docking and molecular dynamics (MD) simulations, were performed to further elucidate the binding modes of mGluR5 NAMs. As a result, the optimal CoMSIA model obtained shows that cross-validated correlation coefficient *Q*^2^ = 0.70, non-cross-validated correlation coefficient *R*^2^*_ncv_* = 0.89, predicted correlation coefficient *R*^2^*_pre_* = 0.87. Moreover, we found that aryl benzamide series molecules bind as mGluR5 NAMs at Site 1, which consists of amino acids Pro655, Tyr659, Ile625, Ile651, Ile944, Ser658, Ser654, Ser969, Ser965, Ala970, Ala973, Trp945, Phe948, Pro903, Asn907, Val966, Leu904, and Met962. This site is the same as that of other types of NAMs; mGluR5 NAMs are stabilized in the “linear” and “arc” configurations mainly through the H-bonds interactions, π–π stacking interaction with Trp945, and hydrophobic contacts. We hope that the models and information obtained will help understand the interaction mechanism of NAMs and design and optimize NAMs as new types of antidepressants.

## 1. Introduction

Depression has a clinical manifestation of low mood, anhedonia and low energy levels, cognitive impairment, sleep disorders, sexual dysfunction, and gastrointestinal diseases, amongst other symptoms [1]. The combination of the primary and the secondary disabilities of chronic medical disease caused by depression makes it one of the most costly medical burdens in the world [2]. Actually, WHO has predicted that depression will become the second leading cause of disability worldwide by the year 2030 [3]. Therefore, the study of depression has received widespread attention from the whole world.

Depression involves the influence of psychological, genetic, social environment, culture, and physiology [4]. Its etiology and pathogenesis are still unclear, and various mechanisms are still at the stage of hypothesis. Among them, the glutamate contained in the amino acid neurotransmitter hypothesis is considered to be related to the pathophysiological factors of depression [5]. Glutamate is the major excitatory neurotransmitter in the mammalian central nervous system (CNS) [6]. It is not only widely distributed in the CNS but also highly concentrated in the cerebral cortex, hippocampus, and striatum, in which brain regions are all closely associated with mental activity [7]. Glutamate mediates its effects through interaction with ionic and metabotropic glutamate receptors. As a matter of fact, metabotropic glutamate receptors (mGluRs) are a family of 8 subtypes (mGluR1–mGluR8) that belong to the family of G-protein coupled receptors (GPCR) [8]. Among all mGlu receptors, abnormal glutamatergic signaling of mGluR5 leads to a series of CNS-related diseases such as depression, schizophrenia, X-brittle syndrome, and Parkinson’s disease [2]. Therefore, mGluR5 becomes an attractive therapeutic target.

Structurally, mGlu receptors consist of a large amino-terminal venus fly-trap (VFT) extracellular domain (encompassing the orthosteric site), a seven-transmembrane domain (7TMD), and a cysteine-rich domain (CRD) linking the two [9]. Although several crystal structures of the VFT domain are usable for some mGluR subtypes, including mGluR1, mGluR5, mGluR3, and mGluR7, the orthosteric site has proven difficult to develop efficient subtype-selective ortho-ligands due to its highly conserved characteristic [10]. As a result, efforts have been made to identify allosteric modulators that bind in the less conserved 7TMD [10]. Compounds that bind to an allosteric site and do not itself activate the receptor but enhance the receptor response is called a positive allosteric modulator (PAM). Whereas, the compound that binds to allosteric sites and acts as a non-competitive antagonist is referred to as a negative allosteric modulator (NAM) [11]. As NAMs of mGluR5 are potential drugs for the treatment of many psychiatric disorders [8], including depression, the development of NAMs has become another fascinating area of drug research.

Based on the rapid development of molecular biology, chemical informatics, and computer science, drug development has entered a new era, and computer-aided drug design (CADD) came into being. CADD can greatly accelerate the development of new drugs and is more economical. It has, therefore, become more widely used in the pharmaceutical industry. In this study, we employed a set of CADD methods, including the establishment of a three-dimensional quantitative structure-activity relationship (3D-QSAR) model using both the comparative molecular field analysis (CoMFA) [12] and comparative molecular similarity indices analysis (CoMSIA) [13] methods for the study of a series of newly synthesized [14,15,16] aryl benzamide NAMs of mGluR5. In addition, we also used molecular docking methods to verify the accuracy and stability of the 3D-QSAR model and then used molecular dynamics (MD) to complement the results of molecular docking. These two methods can help us better explore the possible binding conformation between antagonists and target proteins, and thus reveal the corresponding interaction mechanism. We hope that the developed 3D-QSAR model will provide theoretical guidance for the design and development of NAMs of mGluR5 as antidepressants.

## 2. Results

### 2.1. 3D-QSAR Statistical Analysis

To derive a reliable model, three alignment methods are adopted, namely, alignment-I, -II, and -III. The optimal results based on partial least squares (PLS) statistical analysis are shown in Table 1.

In general, $Q^{2}$ > 0.50 [17] is one of the best parameters to evaluate whether a good 3D-QSAR model is acceptable. According to statistical analysis results of PLS, in all CoMFA and CoMSIA models, both alignment-II and -III methods lead to statistically unacceptable models with $Q^{2}$ < 0.5, but alignment-I obtains a more significant statistical value ($Q^{2}$ = 0.47 and 0.70 for the optimal CoMFA and CoMSIA models, respectively). Therefore, only the optimal 3D-QSAR models obtained based on the alignment-I approach are analyzed further presently. As shown in Table 1, the optimal CoMFA model established by using the steric and electrostatic field descriptors has a $Q^{2}$ value of 0.47, which does not meet the requirements, indicating relatively poor internal predictability. However, the optimal CoMSIA model that was constructed from space, electrostatic, hydrophobic, hydrogen bonding (H-bond) donor, and H-bond acceptor descriptors has a $Q^{2}$ value of 0.70, a high $R_{ncv}^{2}$ value of 0.89, a high F value of 120.28, and a low SEE value of 0.22 with 10 OPN, proving its good internal predictability. The relative contributions of steric, electrostatic, hydrophobic, H-bond donor, and H-bond acceptor fields are 14.0%, 29.1%, 34.5%, 2.5%, and 19.9%, respectively. Among them, the contribution of the hydrophobic field ranks first, indicating that the hydrophobic interaction plays a vital role in enhancing the inhibitory activity. In addition, a testing set composed of 24 compounds is used in this study to verify the robustness of the models. Generally speaking, the criterion of $R_{pre}^{2}>0.6$ and $\left(R_{ncv}^{2}-R_{pre}^{2}\right)/R_{ncv}^{2}\leq 0.1$ indicates that the model has a good external prediction ability. While for the optimal CoMSIA model, it meets quite well this criterion with a high value of $R_{pre}^{2}=0.87$ and small difference of $\left(R_{ncv}^{2}-R_{pre}^{2}\right)/R_{ncv}^{2}\;$(= 0.02), indicating its proper external prediction ability.

**Figure 1:**
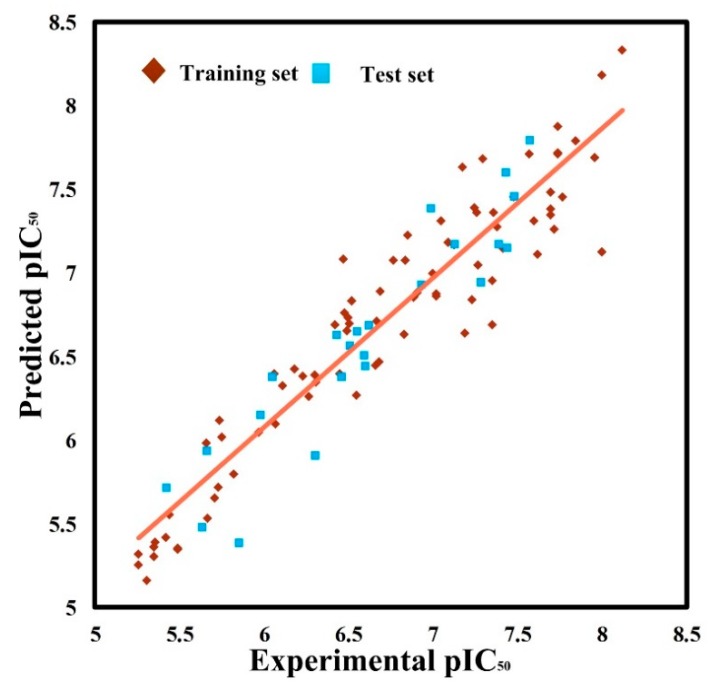
Scatterplot of predicted and experimental ${\mathrm{pIC}}_{50}$ values for the training (brown diamonds) and testing (blue squares) compounds from the optimal comparative molecular similarity indices analysis (CoMSIA) model. The solid line is the regression line for the predicted and experimental ${\mathrm{pIC}}_{50}$ values among the training and testing sets.

In summary, in alignment-I, the optimal CoMSIA model exhibits good internal and external prediction abilities. Its correlation with the entire dataset is given in the way of a scatterplot, as shown in Figure 1. It can be seen that all data points are evenly distributed around the regression line, indicating a good correlation between predicted and experimental biological activity data (${\mathrm{pIC}}_{50}$).

### 2.2. CoMSIA Graphical Interpretation

**Figure 2:**
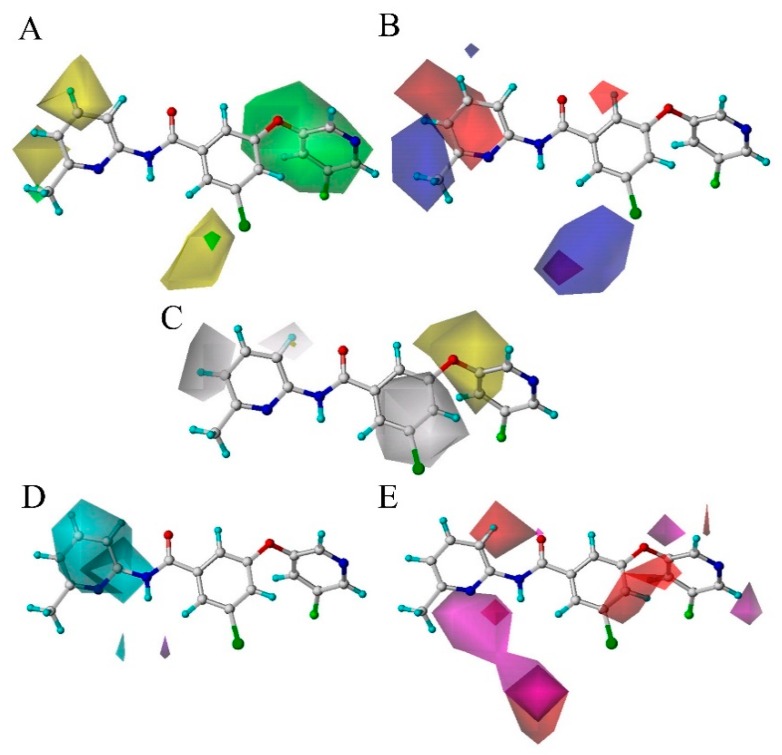
CoMSIA StDev*-Coeff contour map. (**A**) Steric contour map: the green contours represent regions where bulky groups increase the activity, the yellow contours represent regions where bulky groups decrease the activity; (**B**) electrostatic contour map: the blue contours indicate regions where electropositive features promote the activity, the red contours indicate regions where electronegative features promote the activity; (**C**) hydrophobic contour map: the white contours indicate regions where hydrophobic groups are detrimental for the activity, the yellow contours indicate regions where hydrophobic groups are beneficial for the activity; (**D**) H-bond donor contour map: the cyan contours represent regions where H-bond donors promote the activity, the purple contours represent regions where H-bond donors demote the activity; (**E**) H-bond acceptor contour map: the purple contours represent the regions where H-bond acceptors are beneficial for the activity, while the red contours represent the regions where H-bond acceptors are detrimental to the activity.

In this section, the optimal CoMSIA model is selected to construct the StDev*-Coeff contour maps for analysis of the field effects of the ligand molecules. To better illustrate and analyze the results of the Stdev*-Coeff contour maps, the most active molecule **74** from the entire dataset is superimposed with five fields (steric field, electrostatic field, hydrophobic field, H-bond donor field, H-bond acceptor field), as shown in Figure 2.

Figure 2A shows the effect of the sterically hindered field on the molecular activity, where the green cloud indicates that the introduction of large substituents in this region will lead to the increase of the activity of the antagonist, which can be verified from the sample dataset in Table S1 of Supplementary Material. For example, compounds **6** (${\mathrm{pIC}}_{50}=7.57$) and **8** (${\mathrm{pIC}}_{50}=6.49$) have similar structures. However, compound **6** has a greater steric hindrance than compound **8** on the substituent at position 20 of ring A, resulting in an increase in antagonistic activity. Further, the brownish-yellow cloud (20% contribution) indicates that the introduction of a sterically hindered group (i.e., a large substituent) in this region will decrease the antagonistic activity. For example, there is also a yellow cloud around ring C, which also shows that the addition of a substituent with a large steric hindrance will be detrimental to the antagonistic activity. As for molecules **41** (${\mathrm{pIC}}_{50}=6.67$) and **42** (${\mathrm{pIC}}_{50}=5.98$) as compound **42** has an extra fluorine group at position 4 of ring C than compound **41**, the molecular activity is lowered.

Figure 2B depicts the influence of the electrostatic substituent on the potency of the molecules, where a positive charge group at the blue cloud (80% contribution) position or a negative charge group at the red cloud (20% contribution) position will facilitate the activity. As seen from the figure, the blue profile at the R2 substituent of ring C indicates that a positively charged group at that site shall facilitate the activity. This is consistent with the experimental results for a compound activity sequence of **35** (having fewer fluorine atoms on the R2 substituent) > **36**. Whereas, a red profile is observed at positions 4 and 5 of ring C, which indicates the favor of negatively charged groups for improving the activity, for example, compound **40** with a Cl atom at position 5 (${\mathrm{pIC}}_{50}=7.38$) is more potent than compound **41** (${\mathrm{pIC}}_{50}=6.67$) with a methyl substituent in the same region. This can be explained by the existence of an electron-accepting F atom in the substituent at 4-position.

Regarding the equipotential map of the hydrophobic field (as shown in Figure 2C), the white contours indicate the position where the hydrophilic group is favored, and the yellow outline indicates the region of the preferred hydrophobic group. From Figure 2C, we find that there is a large yellow cloud around ring A, which means that the hydrophobic group in the vicinity is advantageous for antagonistic activity. This may explain why compound **29** (${\mathrm{pIC}}_{50}=6.62$) having more hydrophobic groups (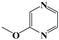) at these regions exhibits higher mGluR5 antagonist activity than compound **94** (${\mathrm{pIC}}_{50}=6.05$). In addition, a large gray cloud at ring B reveals that the hydrophobic groups herein reduce the activity of the molecule. This is well illustrated by the higher activities of molecule **9** (${\mathrm{pIC}}_{50}=7.69$) (without any substituent) than molecule **10** (${\mathrm{pIC}}_{50}=5.85$) with a methyl group at the same position.

Figure 2D is an equipotential map of the hydrogen bond donor field for compound **74**, where the cyan cloud represents the desired while the magenta cloud represents the undesired regions of the H-bond donor, respectively. A cyan cloud is observed covering the NH of the amide group, indicating that NH plays a significant role in donating H near ring C to form a H-bond interaction with NAMs. In addition, a large cyan cloud near ring C indicates that the presence of a hydrogen bond donor substituent will increase the antagonistic activity. This fits well with the experimental results that compound **42** (${\mathrm{pIC}}_{50}=5.98$) with a -CF_3_ group at ring C is less active than compound **39** (${\mathrm{pIC}}_{50}=7.02$) without any group at the same position. Furthermore, almost no purple cloud is found in Figure 2D, which means that unfavorable hydrogen bond donor interactions can be negligible.

Figure 2E is the equipotential map of the hydrogen bond acceptor field. The favorable and unfavorable regions of the H-bond acceptor substituent are respectively represented by the purple (80% contribution) and red (20% contribution) clouds. As seen from the figure, a purple cloud is observed below ring C, which is consistent with the N atom at the 1-position as the H-bond acceptor. In addition, a red cloud is observed near ring B suggesting that the introduction of an H-bond acceptor group in this area would adversely affect the molecule, which is in agreement with the fact that compound **23** is less potent than compound **25**. The reason is that ring B of compound **23** has an F atom as a substituent, while the ring B of compound **25** has a methyl group as a substituent, respectively.

**Figure 3:**
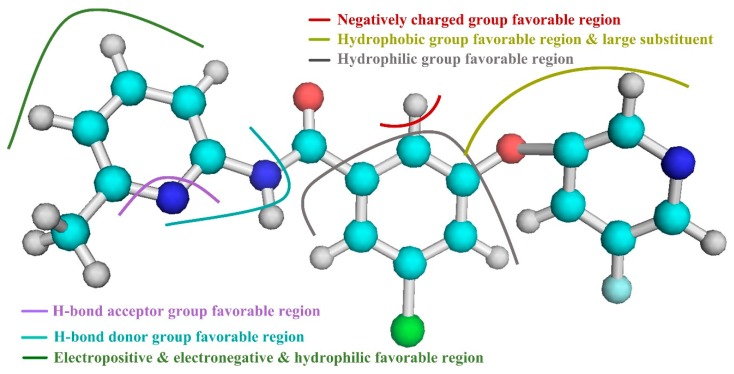
Key structural features of aryl benzamide-based mGluR5 negative allosteric modulators (NAMs) molecules that impact their inhibitory effects.

Based on the above results of the 3D-QSAR study, we are able to determine several of the structural requirements described above for observed inhibitory activity (as shown in Figure 3):

(1) For ring A and the ether bond, the large sterically hindered group and the hydrophobic group contribute to the improvement of molecular activity.

(2) For ring B, away from the R1 substituent, the negatively charged group facilitates the increase in molecular activity. In addition, ring B is close to the R1 substituent, the hydrophilic group is beneficial to the improvement of molecular activity.

(3) For the portion of ring A away from the R2 substituent, and around the N atom of the molecular skeleton, the hydrogen bond donor group facilitates the improvement of molecular activity.

(4) For ring A near the R2 substituent, the portion adjacent to the molecular skeleton, the hydrogen bond acceptor group facilitates the improvement of molecular activity.

(5) For ring A, away from the R2 substituent and the molecular skeleton, the negatively charged group and the hydrophilic group contribute to the improvement of the molecular activity. In addition, ring A is close to the R2 substituent, the positively charged group is advantageous for the improvement of molecular activity away from the molecular skeleton.

### 2.3. Molecular Docking Studies

**Figure 4:**
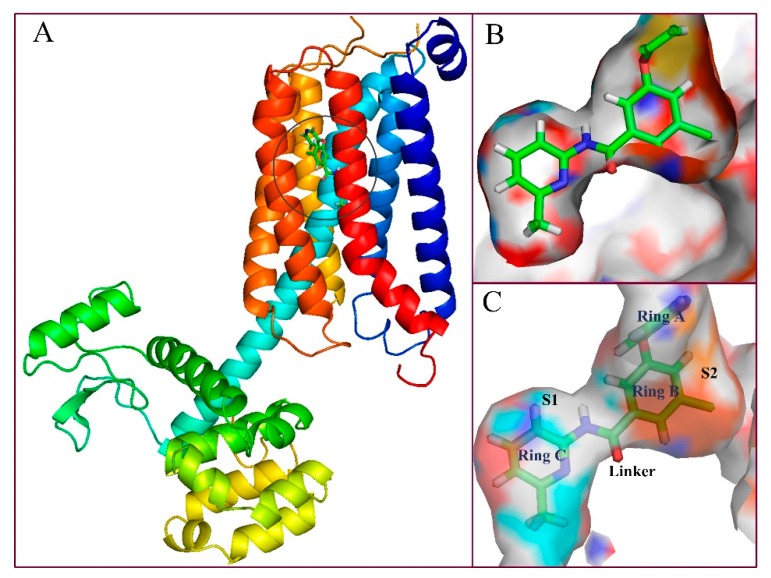
(**A**) Binding pattern of compound **74** with mGluR5; (**B**) and (**C**) depict surface structures and perspective views of the cavity of the binding sites.

Molecular docking is based on the structure of receptor proteins and ligand small molecules, in which chemical statistics are used to simulate the three-dimensional structure of compounds and the interaction between molecules, and in this way, the binding mode between receptors and ligands may be identified. Presently, a computational docking study was performed, and the resultant binding pattern using the most active molecule **74** docked into the human mGluR5 receptor as an example is shown in Figure 4.

As shown in Figure 4A, the sequence of mGluR5 receptor consists essentially of 7 (TM1-TM7) transmembrane helices, in which the binding site of the ligand is embedded in the middle portion of the helical domain. In fact, the binding cavity is an almost closed space resembling a “bone” shape, which is mainly composed of two sub-pockets (represented by S1 and S2 in the present work) and an intermediate connecting region (Linker, Figure 4B). Wherein ring C is located in sub-pocket S1, rings A and B are co-located in another sub-pocket S2, and the molecular skeleton peptide bond is located in the linker of the “bone” site. It can be clearly observed that the area of the cavity where ring C is located is relatively narrow and thus tightly confines this ring in the pocket, which is consistent with the results obtained from the previous QSAR equipotential map (Figure 2A), where a large brownish-yellow cloud is observed around ring C, indicating that the large sterically hindered group is not conducive to the increase in activity. Comparatively, the space of sub-pocket S2 where rings A, B, and the ether bond are located is much wider, which is also consistent with the green contour present around rings A and B in Figure 2A. Therefore, both the 3D-QSAR and docking results indicate that the large sterically hindered groups within the space of sub-pocket S2 where rings A, B, and the ether bond are located contribute to the increase of molecular activity, whereas bulky substituents around ring C disfavor the activity.

**Figure 5:**
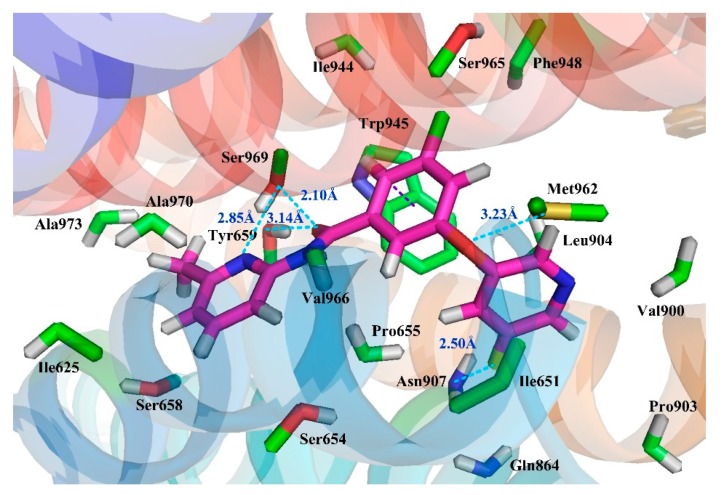
Putative interaction between compound **74** and mGluR5. The amino acid residues are shown by the stick model, in which the molecular carbon and the amino acid residue carbon are represented by pink and green, respectively; the N, O, S, F atoms are represented by navy blue, red, orange, and yellow, respectively; the H-bond force and the π–π stack are represented by blue and purple dotted lines, respectively.

To reveal the binding conformation of the ligand and the corresponding mechanism of action, we examined the docked complex of the mGluR5 receptor in detail. As shown in Figure 5, the binding cavity is mainly composed of 20 amino acid residues (within the 4.5 Å range of the ligand), i.e., Pro655, Tyr659, Ile625, Ile651, Ile944, Ser658, Ser654, Ser969, Ser965, Ala970, Ala973, Trp945, Phe948, Val900, Pro903, Asn907, Leu904, Gln864, Met962, and Val966. In this binding mode, three significant factors are observed, i.e., hydrophobicity, H-bond, and π–π stacking interactions, which contribute to the intimate interaction of the NAMs with the target receptor. Among the 20 amino acids, hydrophobic residues account for about 65%, which greatly facilitates the induction of the active conformation of the antagonist. In fact, sub-pocket S2 where rings A and B are located is a hydrophobic pocket consisting of 12 key amino acid residues, 9 of which are hydrophobic ones such as Trp945, Leu904, Phe948, and Ile651. The proportion of hydrophobic amino acid residues in the sub-pocket S1 is 50%. These results are also essentially in agreement with the hydrophobic contour distribution of the CoMSIA model (Figure 2C). That is, as shown in this figure, the existence of hydrophobic groups around ring A contributes to the improvement of molecular activity. For example, compounds **74** and **59** are similar in structure. The reason why their inhibitory activities both rank among the top five (compound **74**, pIC_50_ = 8.13, compound **59**, pIC_50_ = 7.86) depends largely on the presence of a hydrophobic group at ring A. All of these findings indicate that hydrophobicity is the most critical contribution to enhancing the inhibitory activity.

Besides the hydrophobic interactions, the H-bond and π–π stacking interactions also play key roles in the binding of the aryl benzamide derivatives with the receptor. As a matter of fact, as shown in Table 1, the total contribution of hydrogen-bond descriptors of the optimal CoMSIA model reaches 22.4%, clearly underlining the important role of this weak binding force to the potency of mGluR5 NAMs. In line with this, in present docking results (Figure 5), five H-bonds are formed so as to consolidate the molecular conformation. The N atom on ring C forms a H-bond with the side chain of Ser969 (-N···HO-, 2.85 Å), and the linked ether group -O- form another H-bond with the side chain of Met962 (-O···HS-, 3.23 Å). Further, the O atom on peptide bond forms H-bonds with the side chain of Ser969 (-O···-OH, 2.10 Å) and Tyr945 (-O···-OH, 3.14 Å), respectively. The F atom on ring A also form an H-bond with the side chain of Asn907 (-F···-NH, 2.50 Å). This is in good agreement with the presence of large cyan and small red contours above the link chain in Figure 2D, E). For example, among these five H-bonds, the F atom on ring A and the O atom on the ether bond as the H-bond acceptor substituents are favorable for the inhibitory activity. Furthermore, ring B forms a face-to-face π–π stacking interaction with amino acid residue Trp945, which also helps immobilize the antagonist within the protein cavity. In short, hydrogen bonding and π–π stacking interactions are essential for NAM to exert inhibitory activity.

When anchored in the pocket, molecule **74** is inserted into the transmembrane helices in an “arc”-like conformation (Figure 5), wherein ring C and the amide group form a branch, ring A and the ether group constitute another branch, and ring B constitutes the apex of the “arc,” respectively. The vertex of the “arc” and the branch where ring A is located extend to the hydrophobic pocket, with the branch’s tip fixed by an H-bond formed with residue Asn907. The benzene ring at the apex then further anchors the “arc” by π–π stacking face to face with the residue Trp945. As to the other branch that is composed of ring C and the molecular skeleton peptide bond, it is anchored by an H-bonding interaction with residue Ser969, Tyr659.

In summary, the above hydrophobic interaction, H-bond and π–π stacking interaction play a key role in stabilizing the optimal binding conformation of ligand to mGluR5. In addition, the results of docking and 3D-QSAR models complement and validate each other, providing useful information for the rational design of more effective mGluR5 antagonists in the future.

### 2.4. Molecular Dynamics Studies

**Figure 6:**
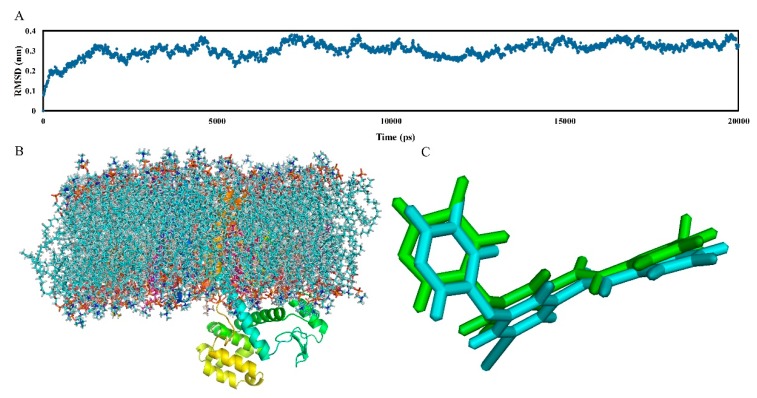
(**A**) Time evolution of the root mean square deviation (RMSD) of the skeletal atom from the initial docking structure during the 20,000 ps molecular dynamic (MD) simulation; (**B**) the structure of the receptor with the docked ligand within the lipid bilayer at the last 1 ns of MD stimulation. Protein, ligand, and lipid molecules are shown as ribbons, lines, and spheres, respectively; (**C**) superposition of the MD structure at the last 1 ns (blue) and initial structure of the docked compound **74** (green).

MD simulation is proof and supplements the 3D-QSAR method and molecular docking analysis, which belongs to the dynamic simulation process. It can not only effectively track and represent the dynamic behavior, but also visually displays the static graphics in the system. In this work, a 20,000 ps MD simulation was performed using the docking complex of the mGluR5 receptor as the starting molecular structure to explore dynamic images of the conformational diversity of ligand-receptor binding. Additionally, to reveal the “positional stability” of the initial complex conformation, the root mean square deviation (RMSD) of the skeletal atoms of the trajectory was calculated, which ends up with a range of 0.25 to 0.40 Å relative to the initial structure of the composite as depicted in Figure 6A. As shown in this figure, the RMSD of the system remains around 0.35 Å after the initial 10,000 ps free balance and is relatively stable throughout the subsequent simulations, indicating a balanced conformation of the complex structure of the docking. In addition, Figure 6C shows the structure of the protein-ligand complex in the MD simulation (blue) superimposed by the initial docking structure (green) in the last 1 ns. As shown in Figure 6C, the structure extracted from the MD simulation agrees well with the docking model of the complex, and both of them adopt have an “arc” conformation. This also proves the rationality of our previous docking model.

**Figure 7:**
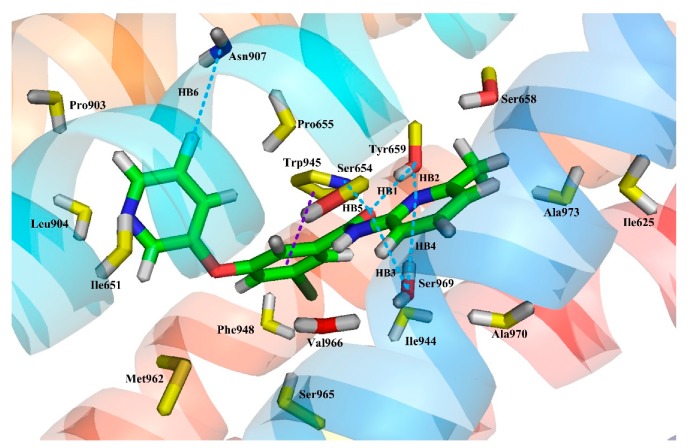
All the binding interactions observed in the last 10 ns MD simulation. Amino acid residues in the active site are represented as sticks; the molecular carbon and the amino acid residue carbon are represented by green and yellow, respectively. The N, O, S, F, and Cl atoms are represented by dark blue, red, orange, cyan, and dark green, respectively. The H-bond and the π–π stacking interactions are represented as blue and purple dashed lines, respectively.

Figure 7 depicts all the binding interactions observed in the last 10 ns MD simulation. As seen from this figure, the binding mode of compound **74** exhibiting in the MD model is almost the same as the docking results in terms of key amino acids and π–π stacking interactions. Specifically, the major amino acids in the docking model constituting the binding cavity are all observed in the MD simulation, including the hydrophobic amino acid residues Ile625, Ile651, Ile944, Ala973, Ala970, Pro655, Pro903, Val966, Phe948, Leu904, Trp945, and Met962, and hydrophilic amino acid residues Ser658, Ser654, Tyr659, Ser969, Ser965, and Asn907. Furthermore, basic face-to-face π–π stacking between ring B and Trp945 is also retained in the MD results.

In addition, because multiple H-bonds are observed in the results of MD simulations, to more precisely quantify H-bonds, the occurrence frequency of H-bonds in the last 10 ns is summarized in Table 2. In the MD simulation results, though the composition of H-bonds at different times (ns) changes slightly, as seen from Table 2, the H-bond interactions in the MD results are basically consistent with the docking results. Especially, three ammonia acid residues, i.e., Tyr659, Ser969, and Asn907, all participate in the H-bonding interactions in both the docking and MD results. Among them, Tyr659 appears most frequently in the MD results and participates in the formation of two H-bonds, i.e., the H-bond between the O atom on peptide bond and Tyr659 (-O···-OH) and the one between the N atom on ring C and Tyr659 (-O···-N), with an occurrence percentage of 90% and 30%, respectively. In these two H-bonds, the former one also appears in the docking results, indicating its essential role in stabilizing the bioactive conformation of the molecule. For Ser969, it also helps the formation of two H-bonds, which include the H-bond with the O atom on the peptide bond (-O···-OH) and the one with the N atom on ring C (-O···-N)). These two H-bonds are both observed in the docking results, proving the importance of Ser969 in the H-bonding network of mGluR5-aryl benzamide NAMs. As for Asn907, it interacts with the F atom on ring A and in this way forms a H-bond (-F···-N), which helps ring A deviate from the original linear conformation and deflect towards Asn907, thereby forming an arc conformation. It is noteworthy that a H-bond between the O atom on peptide bond and Trp945 (-N···-OH) formed in MD simulation is not observed in the docking results, which may be due to the fact that it is the average property of the ensemble of last 10 ns’ MD simulated bioactive conformations instead of the property at a specific time of the ligand-mGluR5 complex that is depicted in Table 2. Due to the relatively high occurrence percentage (50%) of this H-bond, Trp945 may be also crucial for stabilizing the active conformation of the complex.

All the above may explain why the MD simulated structure of the protein-ligand complex overlaps well with its initial docked structure, with no major difference is observed. In summary, the conformation of the ligand remains stable at the active site of the receptor, and it still exhibits “arc” active conformation under the action of hydrophobicity, H-bond, and π–π stacking interaction.

## 3. Discussion

**Figure 8:**
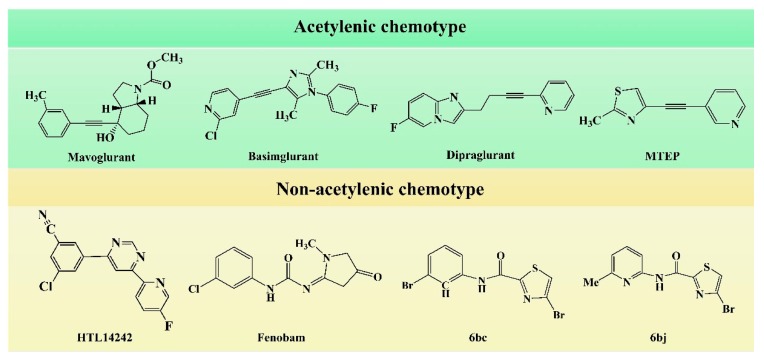
Structure of eight known mGluR5 negative allosteric modulators (NAMs) that have entered and are about to enter clinical trials.

In the early development stage of mGluR5 NAMs, the alkyne subunit in antagonists is regarded as a key structural motif for the molecules to possess a high affinity to mGluR5 [18]. However, an alkyne moiety is metabolically unstable and often cause unfavorable side effects [18]. Therefore, two structural motifs, i.e., amide and urea functional groups, are proposed as the replacements of alkyne linkages in mGluR5 NAMs [18]. So far, altogether eight known mGluR5 NAMs have entered and/or are about to enter clinical trials, i.e., Mavoglurant [19,20,21], Basimglurant [8,21], MTEP [8], HTL14242 [20], Dipraglurant [21], Fenobam [21,22], Thiazole-2-carboxamides (6bc, 6bj) [18]. It is worth mentioning that, for a long time, the important structural information on mGluR5 NAMs was to combine mutation studies with homology modeling and/or a series of ligand analogs. The reason is that the crystal structure of mGluR5-7TM has not been available. It was not until 2014 that Andrew S. Doré [23] obtained the crystal structure of mGluR5-7TM. As, usually, the results of homology modeling are somewhat or slightly different from the actual structures of the target proteins, to further explore the interaction features of various NAMs with mGluR5 receptor, we have summarized the docking/MD results of the above mentioned known mGluR5 NAMs (with molecular structures shown in Figure 8) by using the crystal structure of mGluR5, which is shown in Table 3.

Up to now, two mGluR5 receptor binding regions (the orthosteric binding region in the VFT and the allosteric binding region in the 7TM) have been reported [24]. In fact, we find that the studies on the NAM site almost all focus on the transmembrane allosteric binding region [8,19,20,21,22], which can be explained by the fact that the allosteric binding pocket is less conserved compared to the orthosteric binding region, thus helping the identification of selective ligands [9]. In structure, the 8 antagonists belong to the acetylenic chemotype and non-acetylenic chemotype, as shown in Figure 8. Despite their structural diversity, they bind to the same cavity. By comparing the key amino acids that make up the cavity in Table 2, we find that, as a matter of fact, in the 7TMs of mGluR5 only one binding pocket (Site 1) is identified in charge of interacting with them.

**Figure 9:**
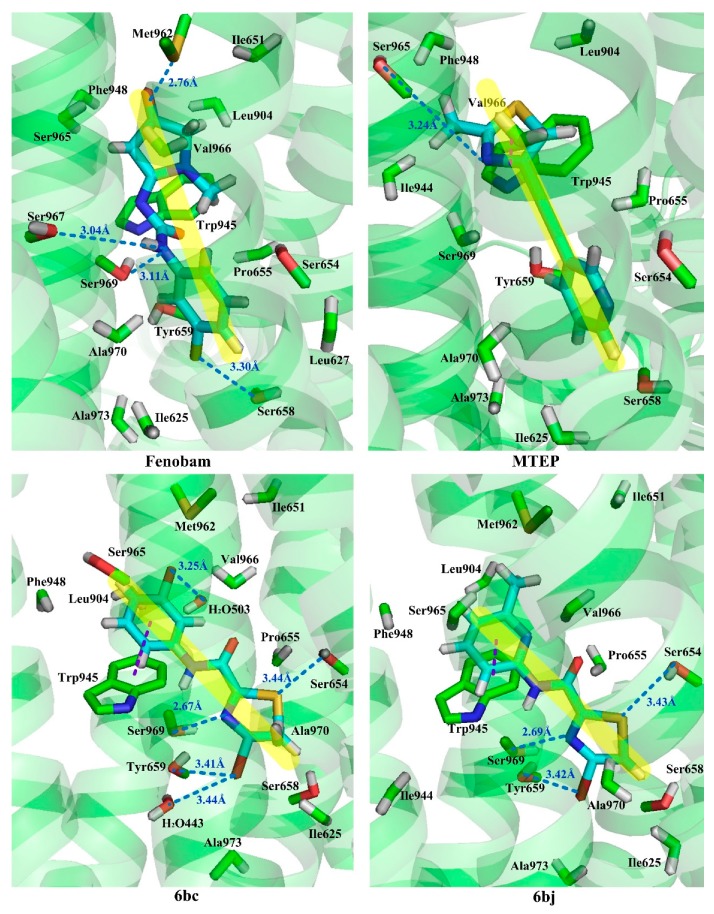
“Linear” binding modes of compounds Fenobam, MTEP, 6bc, and 6bj in the allosteric binding site 1 complexed with mGluR5. The H-bond and π–π stacking interactions are observed as dark blue and purple dashed lines, respectively.

**Figure 10:**
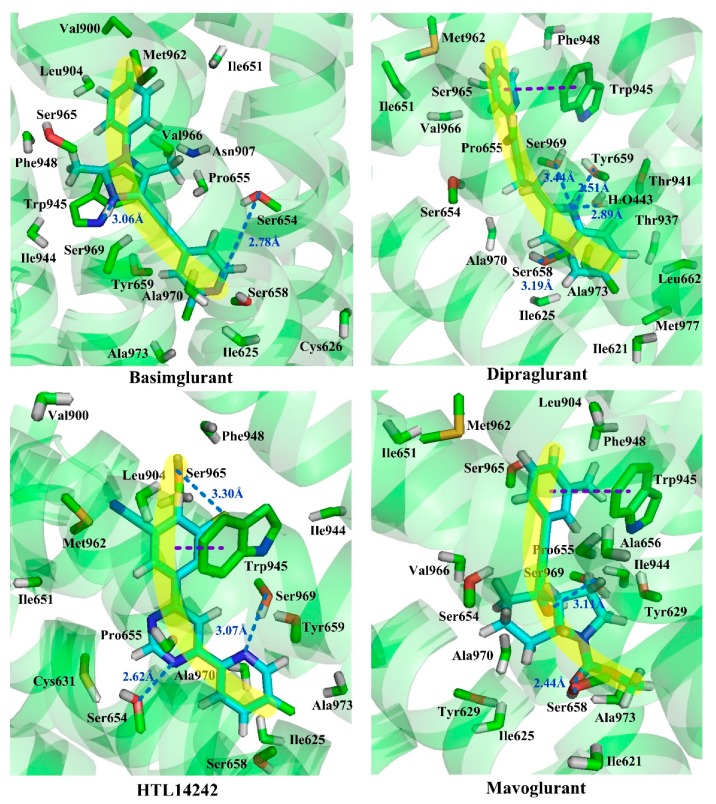
“Arc” binding modes of compounds Mavoglurant, Basimglurant, Dipraglurant, and HTL14242 in the allosteric binding site 1 complexed with mGluR5. The H-bond and π–π stacking interactions are observed as dark blue and purple dashed lines, respectively.

Although they all binding to the same site, these NAMs exhibit different spatial conformations due to their diverse structures. For better comparison of the ligand-receptor binding modes of the antagonists, we performed molecular docking analyses on all above acetylenic and non-acetylenic molecules using PDB 6FFI as mGluR5 template (as same as the present work). Table 4 summarizes the results of these studies. Our docking results show that these molecules do bind to the same cavity in 7TM, and this site consists of two chambers (sub-pocket S1 and sub-pocket S2) and a narrow passage connecting them. As in structure, the skeleton of all NAMs is composed of polycyclic structures, the aromatic ring of the molecules often stretches to one of these two sub-pockets. Factually, by observing our docking results, we can find that the active conformation of all molecules can be divided into two categories, namely “linear” and “arc” configuration, as shown in Figure 9 and Figure 10.

Observation of the docking results reveals that molecules thiazole-2-carboxamide (6bc), (6bj), MTEP and Fenobam are in a “linear” configuration. Takes ligand Thiazole-2-carboxamides (6bj) as an example. Its structural feature is that the body is chain-shaped, and at each end of the chain one aromatic ring is located. Each of the aromatic rings is located in a sub-pocket of the cavity. As shown in Figure 9, in its active conformation, hydrophobic, H-bond, and π–π stacking interactions are involved, wherein hydrophobic interaction of residues Ala973, Ala970, Ile625, Ile944, and Pro655 is identified in the vicinity of the thiazole in the sub-chamber S1. Furthermore, residues Ser654, Tyr659, and Ser969 form a plurality of H-bonds (-O ···S-, 3.43 Å; -O···O-, 3.42 Å; -N···O-, 2.69 Å) with the sulfur, oxygen, and nitrogen atoms in thiazole, respectively in S1. In sub-pocket S2, π–π stacking between pyridyl rings of residue Trp945 and 6bj is observed in a face-to-face manner. Besides, hydrophobic interaction of hydrophobic residues Trp945, Leu904, Val966, and Phe948, is also identified in the vicinity of the pyridyl group in sub-pocket S2. It is the above interaction features that keep 6bj in a linear conformation. In terms of other “linear”-type mGluR5 antagonists, this characteristic is also observed. That is, the molecules are chain-like in skeleton, and there are two aromatic rings at both ends of the chain which enter the two sub-pockets of S1 and S2, respectively when binding to S1. In addition, the skeleton chain linking the two end aromatic rings is comparatively short, generally less than 4 chemical bonds. Thus, it is assumed that NAMs with the above structural characteristics may adopt linear conformations to bind stably with the mGluR5 receptor.

Whereas, molecules Dipalglurant, Mavoglurant, Basimglurant, HTL14242, and Compound **74** are observed in an “arc” configuration in the binding complex. In structure, actually, their skeletons are also chain-like (Figure 10). However, unlike those NAMs with linear active conformation, not only aromatic rings exist at both ends of the chain, but also more than one aromatic ring exists at one end of the chain. In interaction forces, similar to the “linear” configuration, hydrophobic, H-bond, and π–π stacking interactions are also identified contributing to the “arc” configuration. Taking Dipalglurant as an example, where hydrophobic interactions around sub-pockets S1 and S2, and a π–π stacking interaction between the pyridyl group and residue Trp945 in a face-to-face way in S2 are all observed. The essential difference between the “linear” and “arc” configurations is that the polycyclic ring structure in the “arc” configuration provides more opportunity to form H-bond interactions. Actually, in the Dipalglurant-mGlu5 complex, four H-bonds are identified in sub-pocket S1. In addition to three H-bonds formed by the amino acid residues Ser969 (-O···NH-, 3.44 Å), Ser658 (-O···NH-, 3.19 Å), and Tyr659 (-O···N-, 2.51 Å), it also includes a H-bond formed by a water molecule (-O···OH-, 2.89 Å). For compounds Mavoglurant, Basimglurant, HTL 14,242, and Compound **74**, the above characteristics are also observed. Therefore, the molecule is long-chain on the backbone and has one or more aromatic rings at each end of the long chain. The heteroatoms on the polycyclic ring can form a plurality of hydrogen bonds with the surrounding amino acid residues, and the molecule having these characteristics is “arc” conformation.

**Figure 11:**
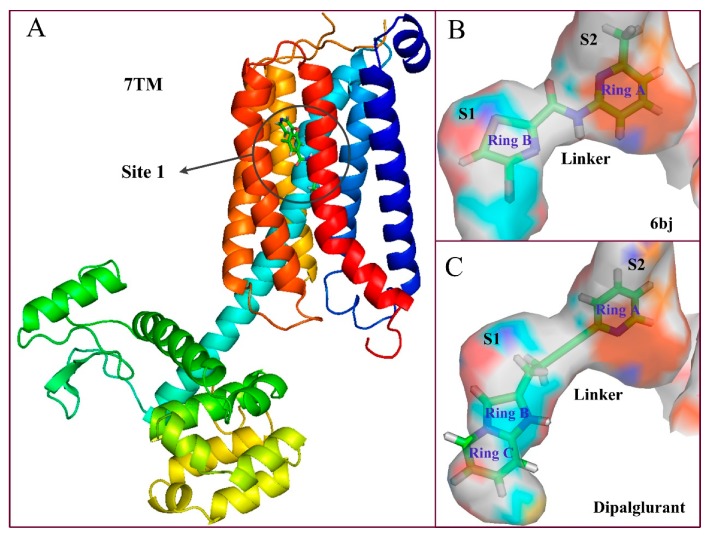
(**A**) NAMs’ binding Site 1. (**B**) The cavity structure of compound 6bj at Site 1. (**C**) The cavity structure of compound Dipalglurant at Site 1.

**Figure 12:**
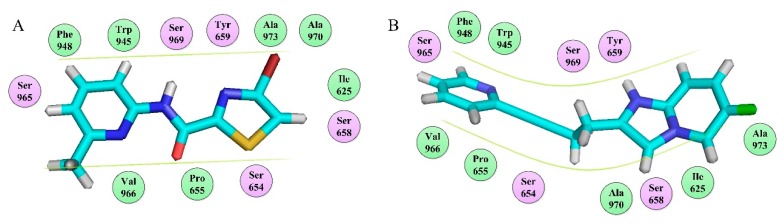
Binding modes of NAMs with mGluR5. (**A**) The “linear” docking modes of compound 6bj. (**B**) The “arc” docking modes of compound Dipalglurant.

To sum up: (1) mGluR5 NAMs all bind at Site 1 (Figure 11A) despite the different spatial conformations of the molecule. The most important residues involved in Site 1 are Tyr659, Pro655, Ser809, and Ser805. (2) This site consists of three main regions including the two chambers (sub-pocket S1 and sub-pocket S2) and a narrow passage connecting them, as shown in Figure 11B, C. (3) The ligand-receptor binding modes are mainly stabilized by the complex interactions including hydrophobic (Pro655, Ile625, Ile651, Ala973, Ala970, Val966, Trp945, and Met962), H-bond (Ser969, Tyr659, Ser658, Ser654, Met962, Asn907) and π–π-stacking (Trp945) interactions. (4) The polycyclic binding ring determines two different molecular active conformations, namely the “linear” and “arc” docking modes, as shown in Figure 12. (5) The aryl benzamide derivatives studied in our work also bind to Site 1 as mGluR5 NAMs, in an “arc” conformation stabilized by hydrophobic, H-bond, and π–π stacking interactions. All these findings, we hope, may be of help for providing insights for the future exploitation of novel mGluR5 NAMs with high inhibitory activities.

Evaluating a 3D-QSAR model depends not only on how well it fits the data but more importantly on how well it predicts the data. Contour maps, molecular docking, and MD simulation results provide guidance for designing domain-specific substituents on compound scaffolds that are critical for improving the inhibitory potency of mGluR5 NAMs. Therefore, presently, to further validate these conclusions, based on the structural skeleton of molecule **74** (as a template), a total of 8 novel aryl benzamide derivatives were designed and their potential activities against mGluR5 were predicted as well adopting the optimal CoMSIA model. Table 5 summarizes the chemical structures of these molecules and their corresponding predicted activities. Unsurprisingly, all of them (NC1~NC8) present higher pIC_50_ values than that of the template compound **74** (pIC_50_ = 8.13), suggesting their great potential to be potent mGluR5 NAMs.

## 4. Materials and Methods

### 4.1. Dataset and Biological Activities

A total of newly synthesized 106 aryl benzamide series of mGluR5 non-competitive antagonists [14,15,16] are adopted to build a dataset for developing a reliable 3D-QSAR model in this study. Their experimental activities are converted into corresponding ${\mathrm{pIC}}_{50}$ ($-\log{\mathrm{IC}}_{50}$) values that are then used as dependent variables for QSAR modeling. The dataset is randomly divided into a training set of 82 compounds and a testing set of 24 compounds at a ratio of approximately 3:1. To ensure that the predictive power of the model be effectively evaluated, the testing set molecules are selected following the rules: (1) their ${\mathrm{pIC}}_{50}$ values are randomly but evenly distributed over the entire range of test values; (2) their structure covers the largest possible diversity of the dataset, so the derived models can represent the true characteristics of all compounds from both the biological activity and the molecular structures. The information of all 106 molecules is provided in the support information in Table S1.

### 4.2. Molecular Modeling and Alignment

**Figure 13:**
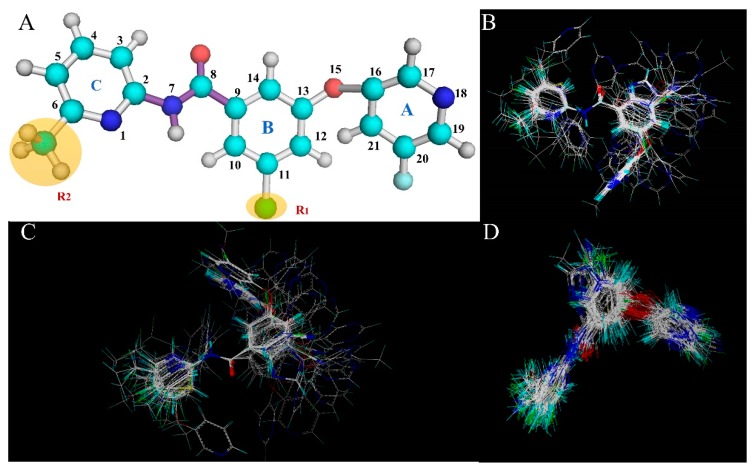
Molecular alignment of all compounds in the dataset. (**A**) The most effective compound **74** is used as a template. The common structure is marked by the purple stick. The molecule is represented by a rod model in which the molecules C, H, N, O, Cl, and F are represented by white, cyan, navy blue, red, green, and blue, respectively. Alignment-I, -II, and -III of all the compounds are shown in panels (**B**), (**C**), and (**D**), respectively.

Before 3D-QSAR modeling, it is necessary to perform conformational optimization for the chemicals from the sample dataset. For generating the optimal 3D-QSAR model, three different alignment rules are adopted, i.e., alignment-I, -II, and -III. The first alignment strategy is an atom-based approach, where all optimized molecules are stacked into the template using the Align Database module in SYBYL. The most potent antagonist (molecule **74**) is selected as a template for molecular alignment to construct all 3D-QSAR statistical models (Figure 13B). Alignment-II (Figure 13C) is a receptor-based approach, which is generated based on the active conformation of template compound **74** that is derived from the docking simulation. The third alignment rule (Figure 13D) is still a receptor-based approach. In this method, all active conformations of the whole dataset molecules are first extracted from the dock studies, and then subjected to the process of alignment-I, i.e., the conformation of molecule **74** is also chosen as the template molecule to be superposed by all other molecules using receptor-based conformations. Figure 13A depicts the common skeleton (peptide bond) of compound **74** in the molecular superposition which is shown by the purple stick. In addition, the superposed models built based on alignment-I, II, and III are shown in Figure 13B, C, D, respectively.

Then the modeling process is performed. The partial atomic charge is calculated using the Gasteiger–Hückel method [25], and the energy is minimized using the Tripos molecular mechanics field and the Powell conjugate gradient minimization algorithm [26], where the convergence criteria are set to 0.05 $\mathrm{kcal}\cdot {\mathrm{mol}}^{-1}\cdot {Å}^{-1}$ to ensure the stability of the conformation.

### 4.3. CoMFA and CoMSIA Studies

After molecular stacking, all molecules are placed in a 3D lattice with a grid spacing of 2 Å. Two different 3D-QSAR methods, CoMFA and CoMSIA, are used to compare and analyze the quantitative relationships between the three-dimensional structural features and the biological activity of the molecules.

The CoMFA field is analyzed by using a sp^3^ C atom probe with a formal charge of +1.0 for each lattice point and a Van der Waals (VdW) radius of 1.52 Å [27]. The stereo and electrostatic fields are calculated using the CoMFA standard method with energy cut-off values of 30.0 kcal/mol [28]. CoMSIA is an extension of CoMFA, and the CoMSIA similarity index descriptor is derived using the same grid box as in the CoMFA calculation. Five different similar descriptors for steric, electrostatic, hydrophobic, and H-bond donors and acceptors are calculated by using a probe atomic charge +1.0, radius 1.0 Å, and hydrophobicity +1.0. The Gaussian function is used to estimate the mutual distance between each molecular atom and the probe atom, and there is no cut-off limit in the CoMSIA study.

A statistically significant 3D-QSAR model is obtained by using PLS regression analysis, where the relationship between the experimental inhibitory activity ${\mathrm{pIC}}_{50}$ and CoMFA and CoMSIA descriptors are analyzed [29,30]. In the PLS analysis, we use the leave-one-out method to evaluate the reliability of the model by calculating the conventional correlation coefficient ($Q^{2}$), the standard prediction error (SEP), and the optimum number of components (OPN). Using the molecules of all training sets in combination with OPN, Pearson coefficients ($R_{ncv}^{2}$), standard error of estimate (SEE), and F value are calculated by non-cross-validation analysis [31]. Among them, $Q^{2}$ and $R_{ncv}^{2}$ are used as statistical indicators for the model prediction ability. An independent testing set is used to evaluate the predictive power of the CoMFA/CoMSIA methods. The CoMFA/CoMSIA similarity index, $Q^{2}$ and predicted $R^{2}$ ($R_{pre}^{2}$) values in the above process are calculated. Finally, the CoMFA/CoMSIA results are visually presented in the form of an equipotential map.

### 4.4. Molecular Docking

To study the interaction between the receptor and its ligand, we use Gold (Genetic Optimization for Ligand Docking) 5.1 for molecular docking analysis. It is a flexible docking technique where the protein is considered rigid, and all degrees of freedom of the ligand are explored (i.e., translational, rotational, and conformational) [32]. Using the PDB entry 6FFI protein structure (resolution, 2.6 Å; method, X-ray diffraction) as a template. Then we studied the docking with water molecules but found that there was no force on the water molecules. So, all water molecules are removed from the protein prior to docking, the ligand is also extracted, and hydrogen atoms are added to the receptor. In the docking, we use the corresponding scoring software to judge the complementarity and affinity of the ligand and the receptor. Scores are used to filter all acquired conformations to determine the most reliable conformation.

### 4.5. Molecular Dynamics

To obtain an accurate protein-ligand binding model and to verify the docking results, in this paper, the CHARMM-GUI website (http://www.charmm-gui.org/?doc=input/membrane.bilayer) [33] is used to construct a phospholipid bilayer (POPC) to mimic the cell transmembrane environment at the 7TM site of the mGluR5 protein, and MD simulation is performed using the GROMACS software package [34]. The system of compound **74** with mGluR is solvated by applying TIP3 water model and enough chloride ions are added to reach zero charge [35]. First, the unconstrained energy minimization of the simulated system is performed using the steepest descent algorithm and conjugate gradient algorithm. Then the system is balanced at 300 K via 100 ps MD simulations. Finally, a 20 ns simulation is carried out in 2 fs time steps to maintain the stability of the entire system. Both energy minimization and MD simulation are performed under periodic boundary conditions with a temperature set of 300 K and atmospheric pressure. The electrostatic interactions are calculated using the Particle Mesh Ewald (PME) method [11] during the simulation. The Linear Constraint Solver (LINCS) algorithm [36] constrains the covalent bond involving the H atom. The cutoff distances for calculating the Van der Waals interaction and Coulomb interaction are set to 1.2 and 1.2 nm, respectively. The isothermal compression rate is set to $4.5\times 10^{-5}\;{\mathrm{bar}}^{-1}$ by the Parrinello–Rahman protocol while the pressure is maintained at 1.0 bar [37] and the temperature is kept constant using a Berendsen thermostat.

## 5. Conclusions

In the current work, a comprehensive computer study of a total of 106 newly synthesized mGluR5-NAMs is conducted. Using a combination of 3D-QSAR, molecular docking, and MD simulation, the relationship between the structure of these compounds and their activity, and their interaction with target proteins are explored. Our findings are summarized below:

The optimal CoMSIA model developed is statistically predictable, $Q^{2}$ = 0.70, $R_{ncv}^{2}$ = 0.89, and $R_{pre}^{2}$ = 0.87, demonstrating its significant internal and external predictive capabilities. The key structural determinants that influence the activity of mGluR5-NAM molecules based on the corresponding contour maps are identified.

Furthermore, the aryl benzamide derivatives herein exhibit an approximate “arc” conformation and bind to the Site 1 of the mGluR5 receptor. Hydrophobic, H-bond, and π–π interactions are the main interactions with the cavity.

Through our study, the binding patterns and characteristics of ligand small molecules and metabotropic glutamate receptor proteins are identified, and the mechanism of their binding is explored and interpreted, which provide guidance for the synthesis of new glutamic related neurologic diseases.

## Figures and Tables

**Table 1:** Summary of optimal three-dimensional quantitative structure-activity relationship (3D-QSAR) results based on three different alignment methods.

PLS Statistics	Alignment-I		Alignment-II		Alignment-III	
	CoMFA	CoMSIA	CoMFA	CoMSIA	CoMFA	CoMSIA
$Q^{2}$	0.47	0.70	0.03	0.05	0.35	0.23
$R_{ncv}^{2}$	0.89	0.89	0.24	0.24	0.82	0.78
$R_{pre}^{2}$	0.66	0.87	0.49	0.50	0.99	0.96
SEE	0.29	0.22	0.69	0.68	0.75	0.39
SEP	0.45	0.39	0.69	0.76	0.77	0.82
F	96.44	120.28	14.76	29.71	9.13	30.55
OPN	8	10	2	1	7	7
Field Contribution/%						
Steric	43.5	14.0	47.0	9.0	22.2	10.3
Electrostatic	56.5	29.1	53.0	36.7	77.8	31.7
Hydrophobic	-	34.5		36.9		31.2
H-bond donor	-	2.5		1.3		2.1
H-bond acceptor	-	19.9		16.1		24.7

$Q^{2}$: cross-validated correlation coefficient, SEP: standard error of prediction, SEE: standard error of estimate, OPN: optimum number of components, $R_{ncv}^{2}$: non-cross-validated correlation coefficient, $R_{pre}^{2}$: predicted correlation coefficient, $F=R_{ncv}^{2}/\left(1-R_{ncv}^{2}\right)$.

**Table 2:** The H-bond interactions in MD simulation results within the last 10 ns.

H-Bond Code	Amino Acid Residue	Occurrence Percent of H-Bonds (%)	H-Bond Interactions and Their Constituents
HB1	Tyr659	90	The H-bond between the O atom on the peptide bond and Tyr659 (-O···-OH)
HB2	Tyr659	30	The H-bond between the N atom on ring C and Tyr659 (-O···-N)
HB3	Ser969	70	The H-bond between the O atom on the peptide bond and Ser969 (-O···-OH)
HB4	Ser969	10	The H-bond between the N atom on ring C and Ser969 (-O···-N)
HB5	Trp945	50	The H-bond between the O atom on the peptide bond and Trp945 (-N···-OH)
HB6	Asn907	30	The H-bond between the F atom on ring A and Asn907 (-F···-N)

**Table 3:** The summary of various types of NAMs-mGluR5 docking/MD results.

No	Representative Molecule	Year	Template	Binding Interactions	Crucial Residues	Authors
1	Mavoglurant	2015	mGluR5 (PDB 4OO9)	H-bond	Asn747, Ser805, Ser809, Tyr659, Thr781	Harpsøe et al. [19]
2	Basimglurant, MTEP	2015	mGluR5 (PDB 4OO9)	H-bond	Gly624, Ile625, Gly628, Pro655, Met802, Ser805, Val806, Ser809, Asn747, Tyr659	Feng et al. [8]
3	Thiazole-2-carboxamides (6bc, 6bj)	2016	mGluR5 (PDB 4OO9)	Hydrophobic interaction, H-bond	Pro655, Tyr659, Val806, Ser809, Ala813, Ala810, Ile625	Vu et al. [18]
4	Mavoglurant, HTL14242	2017	mGluR5 (PDB 4OO9, 5CGD)	Hydrophobic interaction, H-bond	Gly624, Ile625, Gly628, Pro655, Ser805, Val806, Ser809, Asn747, Trp785, Tyr659, Thr735	Bian et al. [20]
5	Mavoglurant, Dipraglurant, Basimglurant, Fenobam	2018	mGluR5 (PDB 4OO9, 5CGC, 5CGD)	Hydrophobic interaction, H-bond	Ile625, Pro655, Ala810, Ser654, Ser809, Asn747, Ile651, Tyr659, Ser805, Trp785, Phe788, Leu744, Met802, Val806, Ser658, Val740	Fu et al. [21]
6	Fenobam	2018	mGluR5 (PDB 6FFH)	Hydrophobic interaction, H-bond, π–π stacking	Tyr659, Ser809, Trp785, Phe788, Ser805, Ile651, Leu744, Ile784, Met802, Val806, Pro655	Christopher et al. [22]

**Table 4:** Interaction features of representative mGluR5 NAMs.

No	Category	Representative Molecule	Site Type	Binding Interactions	Binding Conformation
1	Acetylenic	Mavoglurant	Site 1	Hydrophobic, H-bond, π–π interactions	Arc
2	Acetylenic	Basimglurant	Site 1	Hydrophobic, H-bond	Arc
3	Acetylenic	MTEP	Site 1	Hydrophobic, H-bond, π–π interactions	Linear
4	Acetylenic	Dipraglurant	Site 1	Hydrophobic, H-bond, π–π interactions	Arc
5	Non-acetylenic	Thiazole-2-carboxamides (6bc)	Site 1	Hydrophobic, H-bond, π–π interactions	Linear
6	Non-acetylenic	Thiazole-2-carboxamides (6bj)	Site 1	Hydrophobic, H-bond, π–π interactions	Linear
7	Non-acetylenic	Fenobam	Site 1	Hydrophobic, H-bond	Linear
8	Non-acetylenic	HTL14242	Site 1	Hydrophobic, H-bond, π–π interactions	Arc

**Table 5:** Structures and predicted activities of newly designed mGluR5 NAMs.

No	Structures and pIC_50_ Values	No	Structures and pIC_50_ Values
**NC1**	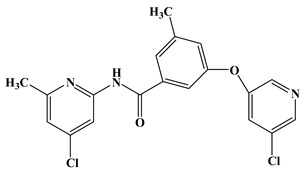 **pIC_50_ = 8.401**	**NC2**	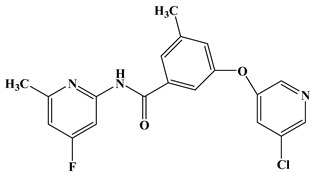 **pIC_50_ = 8.398**
**NC3**	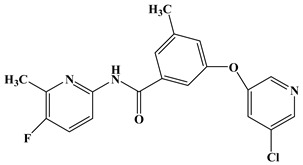 **pIC_50_ = 8.387**	**NC4**	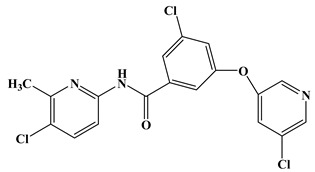 **pIC_50_ = 8.381**
**NC5**	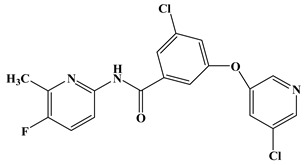 **pIC_50_ = 8.376**	**NC6**	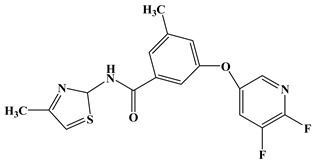 **pIC_50_ = 8.346**
**NC7**	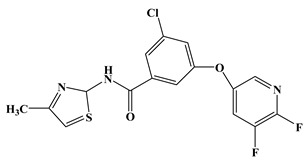 **pIC_50_ = 8.335**	**NC8**	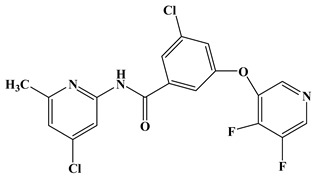 **pIC_50_ = 8.331**

## Supplementary Materials

The following are available online. Table S1: molecular structures and ${\mathrm{pIC}}_{50}$ values of aryl benzamide derivatives.
