# Supplementary material for: Profiling the Structural Determinants of Aryl Benzamide Derivatives as Negative Allosteric Modulators of mGluR5 by In Silico Study
Source: Molecules. 2020 Jan 18;25(2):406. doi: 10.3390/molecules25020406 (PMC7024197; doi:10.3390/molecules25020406)
Supplement: Supplementary file 1 [file molecules-25-00406-s001.pdf]

Table S1. The information of all 106 molecules.

No.	Structure	mGlu5 pIC_{50} (±SEM) %	Glu Max (±SEM) %
1	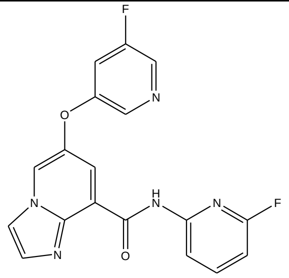	7.42 ± 0.02	1.5 ± 0.2
2	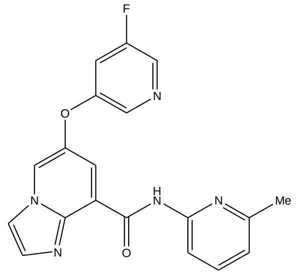	7.18 ± 0.01	1.5 ± 0.2
3	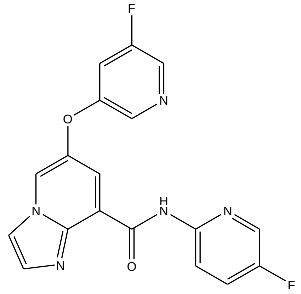	7.69 ± 0.15	1.6 ± 0.1
4	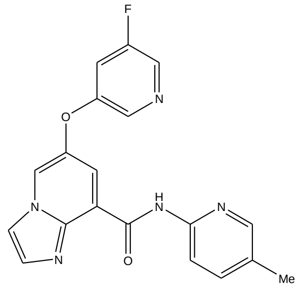	7.13 ± 0.10	1.4 ± 0.2
5	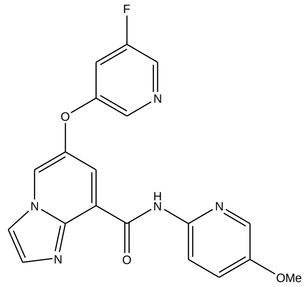	7.26 ± 0.08	1.6 ± 0.2
6	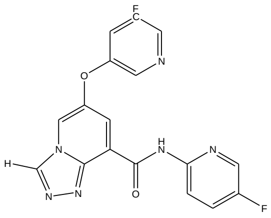	7.57 ± 0.11	2.3 ± 0.1

7	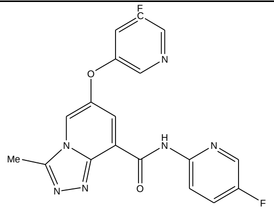	6.43 ± 0.01	2.7 ± 0.9
8	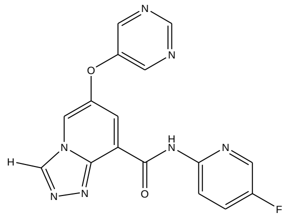	6.49 ± 0.15	1.6 ± 0.2
9	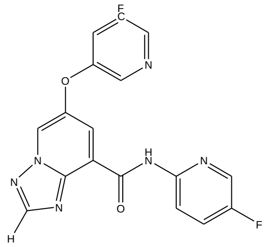	7.69 ± 0.05	1.5 ± 0.2
10	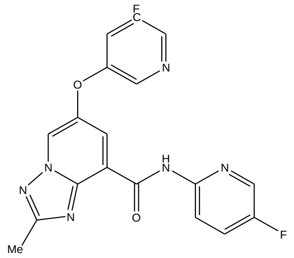	5.85 ± 0.33	3.3 ± 1.6
11	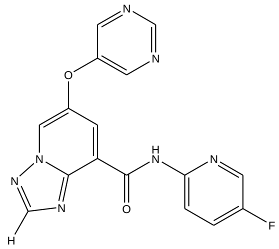	7.13 ± 0.05	1.5 ± 0.2
12	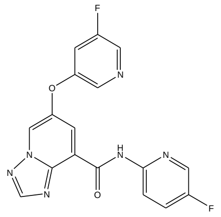	7.69 ± 0.05	1.5 ± 0.2
13	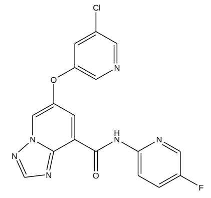	7.05 ± 0.18	1.1 ± 0.2
14	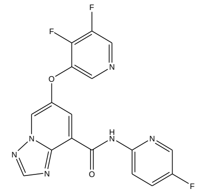	7.39 ± 0.02	2.0 ± 0.1

15	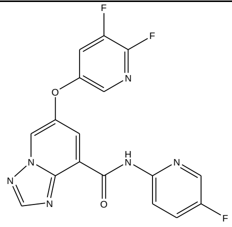	7.25 ± 0.08	1.6 ± 0.3
16	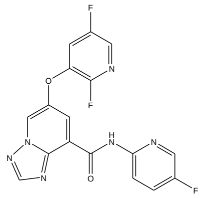	6.93 ± 0.07	2.2 ± 0.6
17	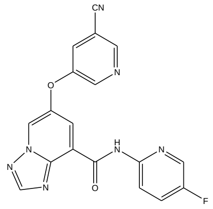	7.36 ± 0.14	2.3 ± 0.4
18	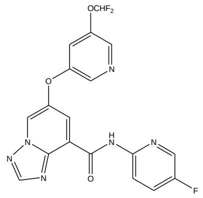	6.89 ± 0.02	1.7 ± 0.2
19	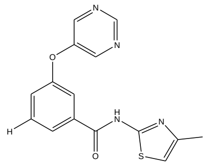	6.69 ± 0.25	1.7 ± 0.3
20	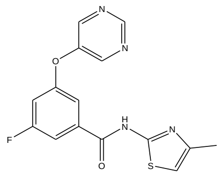	7.62 ± 0.03	1.2 ± 0.1
21	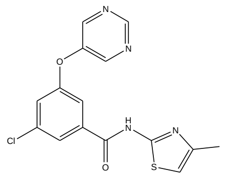	7.94 ± 0.19	1.4 ± 0.2
22	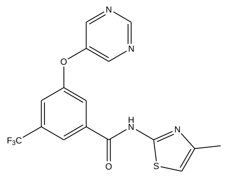	6.55 ± 0.10	1.7 ± 0.2
23	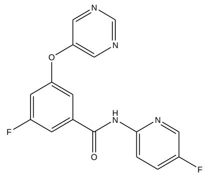	6.52 ± 0.07	1.3 ± 0.3

24	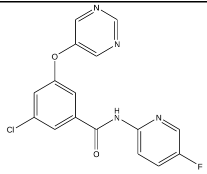	7.49 ± 0.02	1.2 ± 0.2
25	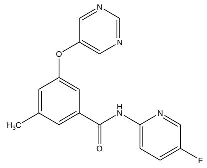	7.72 ± 0.06	0.8 ± 0.2
26	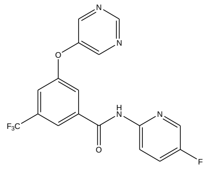	5.26 ± 0.01	3.2 ± 0.3
27	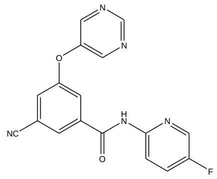	6.50 ± 0.08	1.1 ± 0.4
28	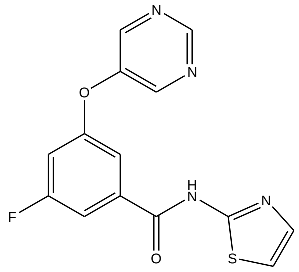	5.75 ± 0.25	3.2 ± 1.2
29	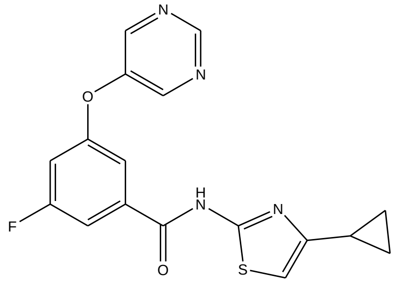	6.62 ± 0.08	1.7 ± 0.3
30	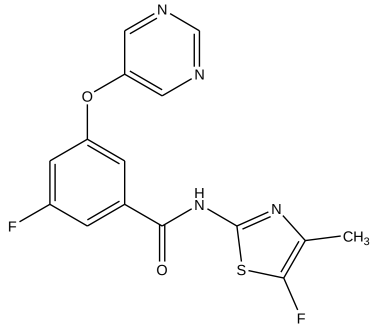	7.27 ± 0.03	1.5 ± 0.1

31	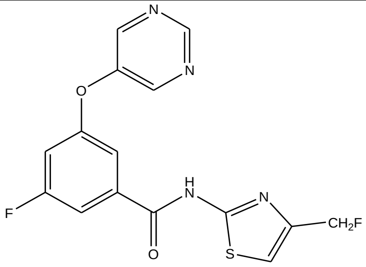	6.66 ± 0.03	1.6 ± 0.4
32	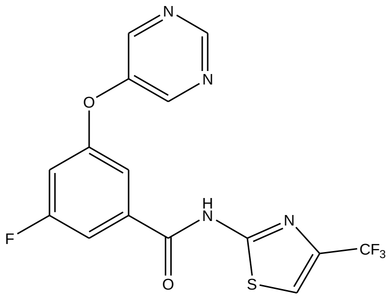	5.66 ± 0.06	1.7 ± 0.4
33	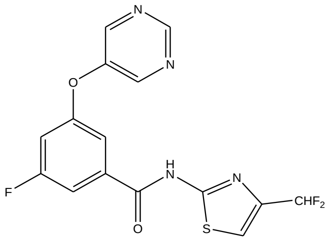	5.49 ± 0.05	1.5 ± 0.5
34	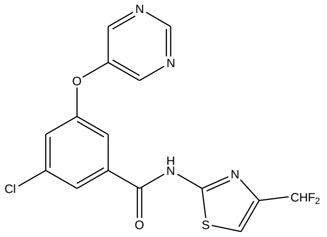	7.09 ± 0.19	1.7 ± 0.2
35	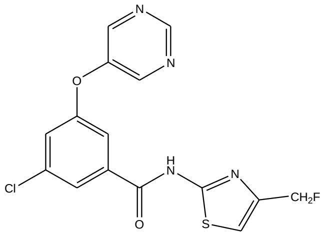	7.44 ± 0.09	1.4 ± 0.2
36	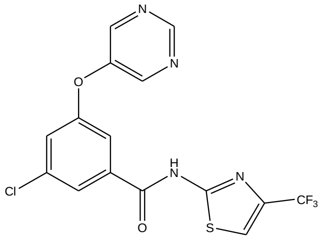	7.01 ± 0.08	1.3 ± 0.2
37	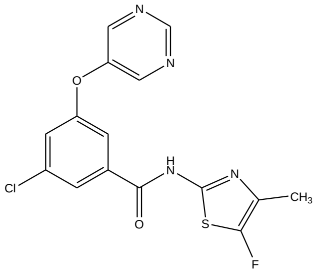	6.51 ± 0.07	1.2 ± 0.2

38	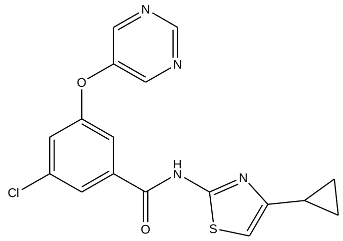	6.68 ± 0.04	1.2 ± 0.4
39	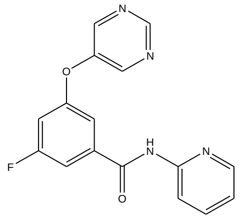	7.02 ± 0.09	1.4 ± 0.2
40	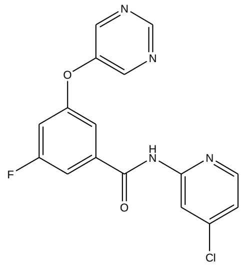	7.38 ± 0.03	1.5 ± 0.1
41	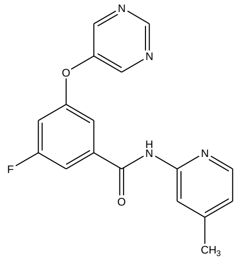	6.67 ± 0.01	1.4 ± 0.2
42	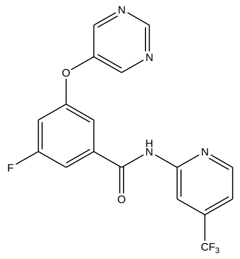	5.98 ± 0.06	1.5 ± 0.3
43	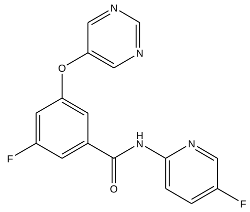	6.52 ± 0.07	1.3 ± 0.3
44	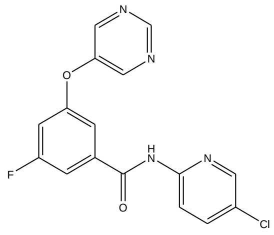	5.97 ± 0.14	2.0 ± 0.4

45	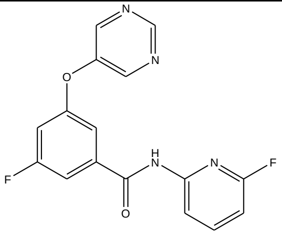	6.59 ± 0.13	1.6 ± 0.2
46	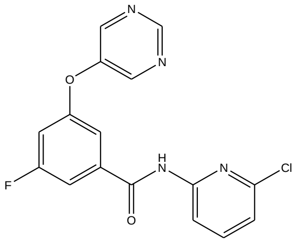	7.35 ± 0.09	1.5 ± 0.5
47	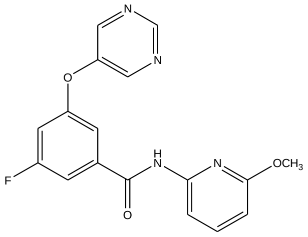	6.18 ± 0.11	1.6 ± 0.3
48	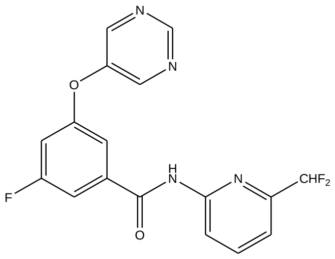	6.83 ± 0.08	1.2 ± 0.4
49	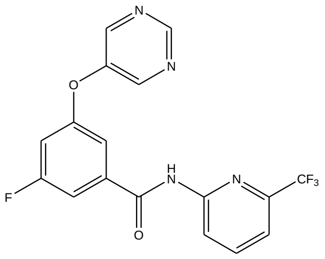	5.64 ± 0.13	3.7 ± 1.3
50	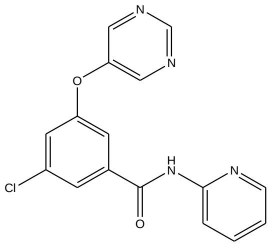	7.77 ± 0.03	1.2 ± 0.4
51	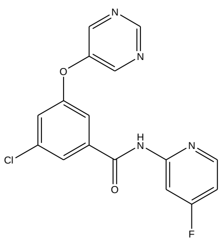	6.47 ± 0.08	1.4 ± 0.2

52	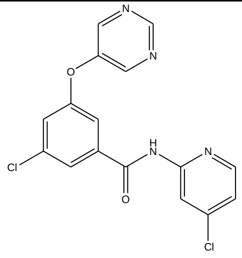	6.77 ± 0.02	1.2 ± 0.2
53	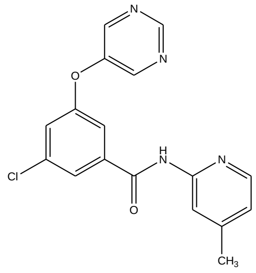	6.85 ± 0.05	1.7 ± 0.2
54	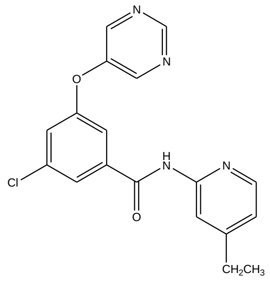	5.35 ± 0.02	2.0 ± 0.7
55	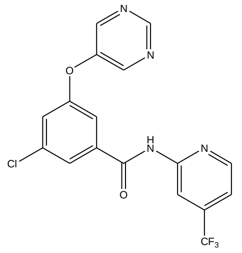	5.36 ± 0.12	2.0 ± 0.0
56	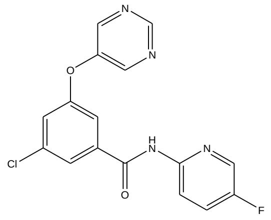	7.49 ± 0.02	1.2 ± 0.2
57	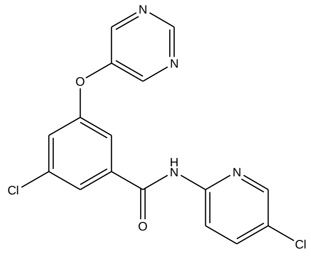	6.99 ± 0.11	1.3 ± 0.4
58	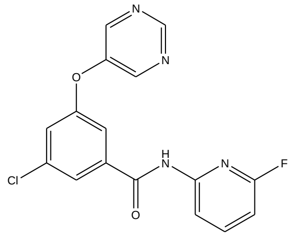	8.00d	0.8d

59	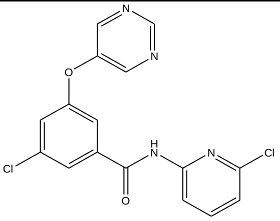	7.86 ± 0.06	1.2 ± 0.3
60	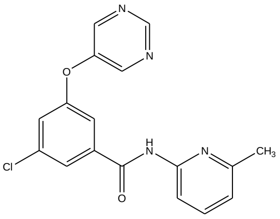	7.75 ± 0.13	1.1 ± 0.3
61	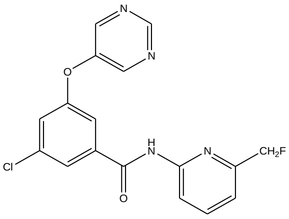	7.74 ± 0.04	1.4 ± 0.4
62	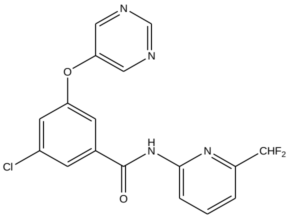	7.44 ± 0.15	1.1 ± 0.4
63	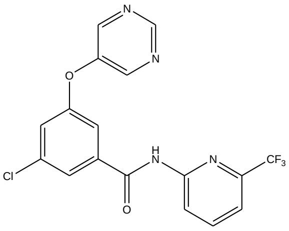	6.51 ± 0.14	1.5 ± 0.2
64	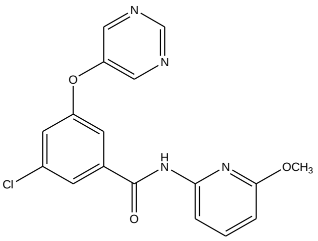	7.27 ± 0.04	1.3 ± 0.2
65	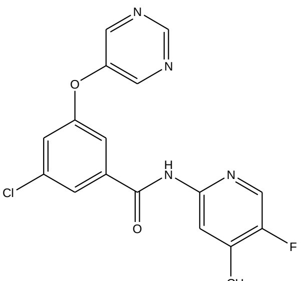	5.49 ± 0.04	10.2 ± 7.7

66	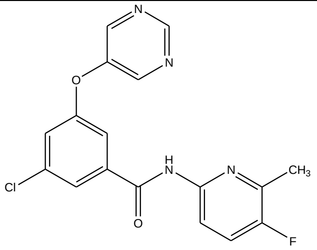	7.30 ± 0.08	1.1 ± 0.3
67	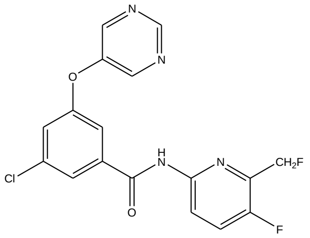	6.84 ± 0.10	1.2 ± 0.3
68	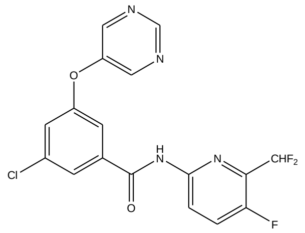	6.30 ± 0.11	1.6 ± 0.3
69	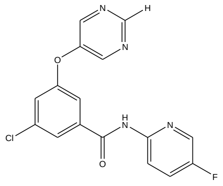	7.49 ± 0.02	1.2 ± 0.2
70	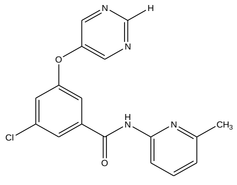	7.75 ± 0.13	1.1 ± 0.3
71	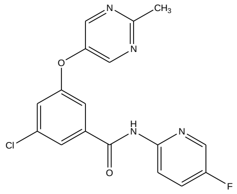	5.35 ± 0.04	2.6 ± 0.1
72	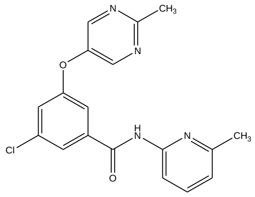	7.02 ± 0.06	1.6 ± 0.2
73	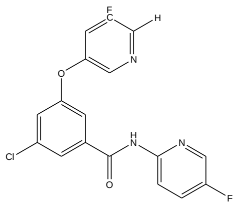	7.61 ± 0.04	1.1 ± 0.1

74	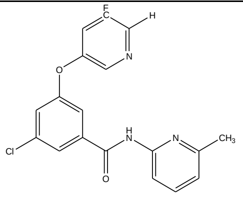	8.13 ± 0.08	1.8 ± 0.4
75	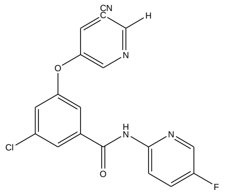	7.57 ± 0.08	1.7 ± 0.2
76	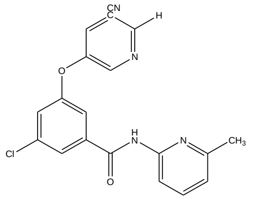	7.99 ± 0.15	1.6 ± 0.1
77	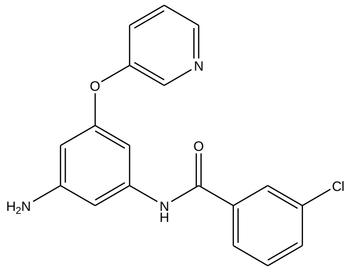	6.55±0.19	1.3±0.2
78	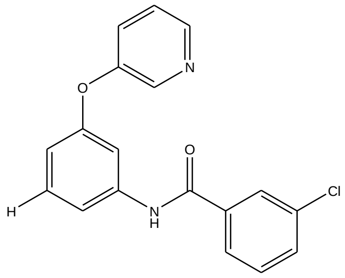	7.09±0.07	1.0±0.2
79	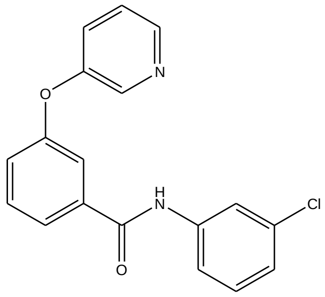	5.71±0.14	1.6±0.4
80	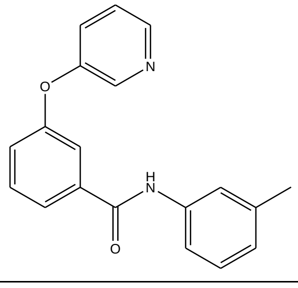	5.66±0.11	1.4±0.1

81	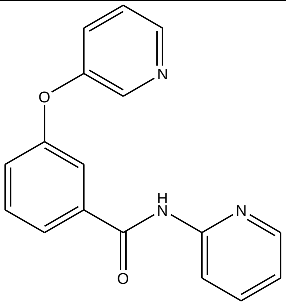	6.07±0.13	1.6±0.6
82	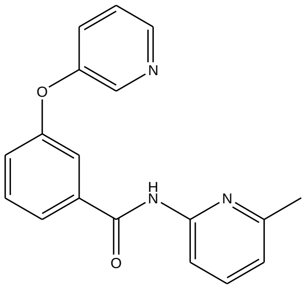	6.45±0.16	1.3±0.4
83	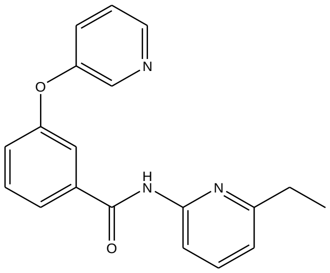	5.73±0.13	1.9±0.2
84	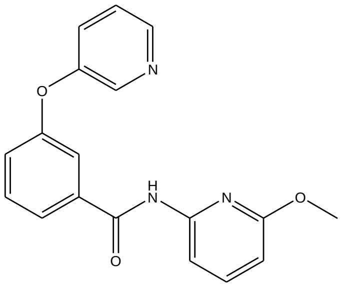	5.44±0.11	3.2±0.9
85	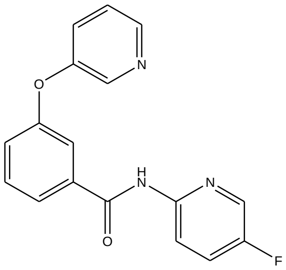	5.17±0.05	6.8±0.9
86	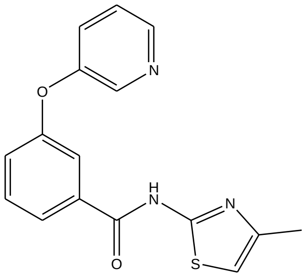	6.11±0.12	1.7±0.2

87	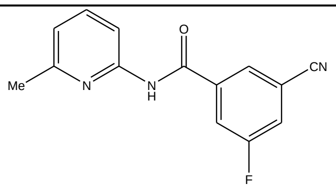	7.18±0.13	1.2 ± 0.7
88	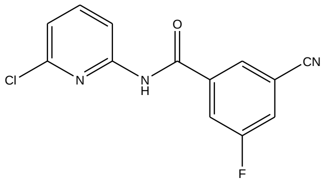	6.60±0.06	2.2 ± 0.2
89	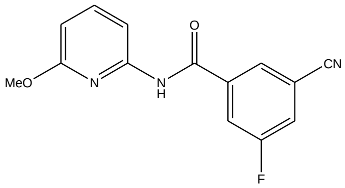	5.82±0.05	17.3 ± 0.4
90	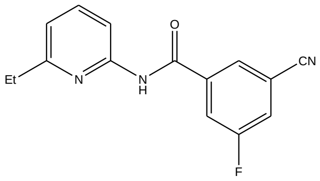	6.29±0.11	1.7 ± 0.5
91	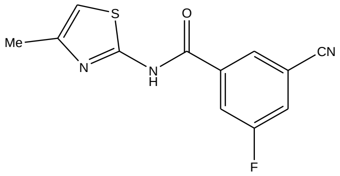	7.22±0.09	1.0 ± 0.3
92	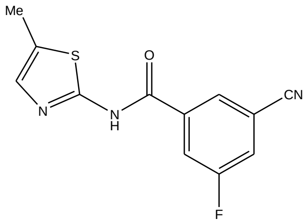	6.06±0.07	17.5 ± 6.8
93	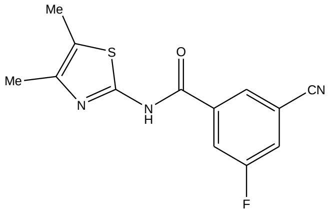	6.22±0.07	2.4 ± 0.4
94	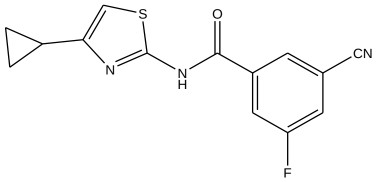	6.05±0.11	1.5 ± 0.5
95	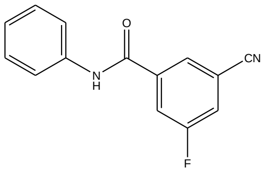	5.26±0.10	12.5 ± 4.7

96	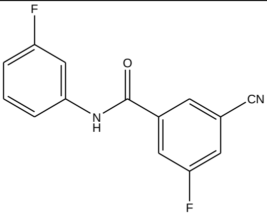	5.67±0.01	22.1 ± 1.1
97	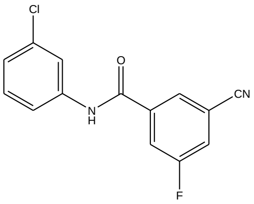	7.35±0.17	0.6 ± 0.2
98	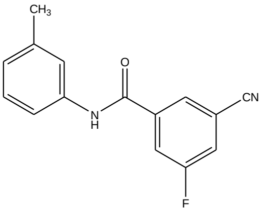	6.91±0.09	2.1 ± 0.2
99	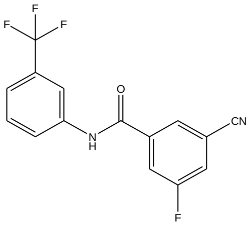	6.27±0.05	0.9 ± 0.3
100	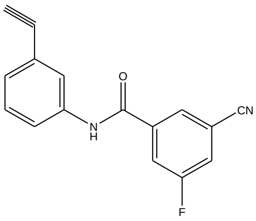	6.31±0.06	1.4 ± 0.3
101	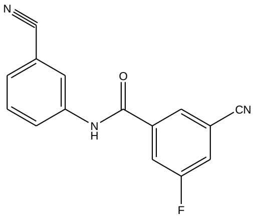	6.48±0.08	2.3 ± 0.3
102	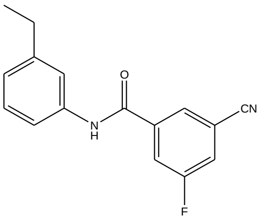	5.31±0.10	2.4 ± 0.2
103	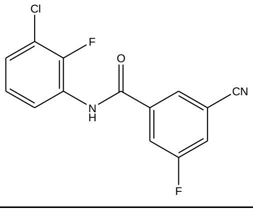	6.46±0.09	2.5 ± 0.5

104	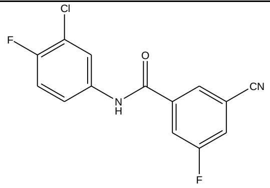	6.42±0.05	43.0 ± 8.0
105	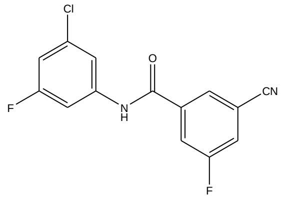	5.74±0.10	38.5 ± 5.2
106	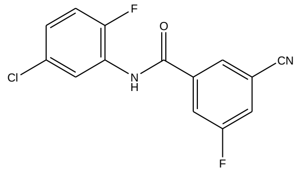	5.42±0.05	29.8 ± 6.6
